# Prevalence, characteristics, and correlates of probable avoidant/restrictive food intake disorder among adult respondents to the National Eating Disorders Association online screen: a cross-sectional study

**DOI:** 10.1186/s40337-023-00939-0

**Published:** 2023-12-04

**Authors:** Laura D’Adamo, Lauren Smolar, Katherine N. Balantekin, C. Barr Taylor, Denise E. Wilfley, Ellen E. Fitzsimmons-Craft

**Affiliations:** 1 Department of Psychological and Brain Sciences and Center for Weight, Eating, and Lifestyle Science (WELL Center), Drexel University, 3201 Chestnut St., Philadelphia, PA 19104 USA; 2 Department of Psychiatry, Washington University School of Medicine, Mailstop 8134-29-2100, 660 S. Euclid Avenue, St. Louis, MO 63110 USA; 3 National Eating Disorders Association, New York, NY USA; 4 Department of Exercise and Nutrition Sciences, School of Public Health and Health Professions, University at Buffalo, Buffalo, NY 14214 USA; 5 Department of Psychiatry and Behavioral Sciences, Stanford University School of Medicine, 401 Quarry Road, Stanford, CA 94305 USA; 6 Center for m2Health, Palo Alto University, 5150 El Camino Real, Los Altos, CA 94022 USA

**Keywords:** Avoidant/restrictive food intake disorder, Feeding and eating disorders, Dietary restriction, Mental health screening

## Abstract

**Background:**

Avoidant/restrictive food intake disorder (ARFID) is a serious, albeit under-researched, feeding or eating disorder. This exploratory study utilized data from adult respondents to the National Eating Disorders Association online eating disorder screen to validate items assessing the presence of ARFID and examine the prevalence, clinical characteristics, and correlates of a positive ARFID screen.

**Methods:**

Among 50,082 adult screen respondents between January 2022 and January 2023, the prevalence of a positive ARFID screen was calculated. Chi-square tests and t-tests compared demographics, eating disorder attitudes and behaviors, suicidal ideation, current eating disorder treatment status, and eating disorder treatment-seeking intentions between respondents with possible ARFID and other eating disorder diagnostic and risk categories. Clinical characteristics of respondents with possible ARFID were also examined.

**Results:**

2378 (4.7%) adult respondents screened positive for ARFID. Respondents with possible ARFID tended to be younger, male, and have lower household income, and were less likely to be White and more likely to be Hispanic/Latino than most other diagnostic/risk groups. They had lower weight/shape concerns and eating disorder behaviors than most other diagnoses and higher BMI than those with AN. 35% reported suicidal ideation, 47% reported intentions to seek treatment for an eating disorder, and 2% reported currently being in treatment. The most common clinical feature of ARFID was lack of interest in eating (80%), followed by food sensory avoidance (55%) and avoidance of food due to fear of aversive consequences (31%).

**Conclusions:**

Findings from this study indicated that ARFID was prevalent among adult screen respondents and more common among individuals who were younger, male, non-White, Hispanic, and lower income relative to those with other eating disorders, at risk for an eating disorder, or at low risk. Individuals with possible ARFID frequently reported suicidal ideation and were rarely in treatment for an eating disorder. Further research is urgently needed to improve advances in the assessment and treatment of ARFID and improve access to care in order to prevent prolonged illness duration.

## Abstract (plain language summary)

This study examined data from adult respondents to a publicly available online eating disorders screen adult to examine the prevalence, clinical characteristics, and correlates of a positive ARFID screen. 4.7% of respondents screened positive for ARFID. A positive ARFID screen was more common among respondents who were younger, male, non-White, Hispanic, and lower income relative to those in other eating disorder diagnostic/risk categories. Respondents with possible ARFID frequently reported suicidal ideation and were rarely in treatment for an eating disorder. Lack of interest in food or eating was the most common clinical feature among those with possible ARFID.

## Background

Avoidant/restrictive food intake disorder (ARFID) is characterized by avoidant or restrictive eating that results in weight loss and/or malnutrition and is not primarily attributable to weight/shape concerns [1]. ARFID is associated with poor quality of life, distress, and functional impairment, and individuals with ARFID often experience gastrointestinal problems, medical consequences, and psychiatric comorbidities [2–8]. Since its addition to the DSM-5 in 2013 [1], ARFID’s prevalence, characteristics, and correlates have remained largely unclear. However, limited research suggests that it may be as common as other feeding or eating disorders, with prevalence estimates ranging from 0.3 to 15.5% in non-clinical samples, 5% to 55.5% in specialized eating disorder service clinics, and 32% to 64% in specialized feeding clinics [8]. ARFID typically emerges during childhood but can affect individuals across the lifespan [1, 9]. Yet, research on ARFID has almost exclusively focused on children and adolescents, with little investigation in adult populations overall and existing adult studies limited to patients in treatment settings [10]. Despite evidence that ARFID is a prevalent and impairing condition, research suggests that it often goes undetected, and no evidence-based treatment recommendations for ARFID currently exist [11, 12]. Thus, additional study into ARFID’s prevalence and characteristics is urgently needed, particularly in under-researched groups including adults and individuals who are not currently in treatment.

Research has supported that ARFID’s presentations are distinct from those of other eating disorders, including anorexia nervosa (AN). Among patients presenting for treatment, those with AN and ARFID have demonstrated similar rates of restrictive eating, weight loss, and malnutrition, but patients with ARFID have shown greater food neophobia, greater fear of choking or vomiting, greater food sensory problems, and lower eating psychopathology [7, 13, 14]. Some studies have also found differences in demographic and clinical characteristics between patients with ARFID and other eating disorders. For instance, patients with ARFID have been found to be younger, have earlier age of eating disorder onset, and be more likely to be male relative to those with other eating disorders [7, 13, 15]. Research has been mixed regarding whether BMI and rates of comorbid anxiety disorders differ between ARFID and AN patients [3, 6, 7, 13, 16–18], as well as whether suicidality differs between patients with ARFID versus other eating disorders [19–21]. Importantly, there are several gaps in this literature that have yet to be explored. Although a few of the aforementioned studies were conducted with adults, the individuals with ARFID in these studies were presenting for eating disorder treatment, and results may not generalize to broader populations of individuals with ARFID. In addition, further research is needed to examine demographic correlates beyond age and gender (e.g., race, ethnicity, sex, income), to more thoroughly explore eating disorder and other forms of psychopathology (e.g., suicidality), and to compare characteristics and correlates of individuals with ARFID to the full spectrum of eating disorders (i.e., instead of just those with AN). Finally, no study to our knowledge has examined whether rates of eating disorder treatment-seeking and current treatment status differ between individuals with ARFID versus those with other eating disorders.

Although the etiology of ARFID is unclear, it is well-documented that ARFID has heterogeneous presentations [9]. Three presentations of restrictive eating in ARFID are currently recognized, which may occur independently or in combination: (1) lack of interest in eating or food, (2) avoidance of eating due to sensory properties of food (e.g., texture, smell, flavor), and (3) avoidance of eating due to fear of aversive consequences from eating (e.g., vomiting, choking, suffocating) [1, 22]. These presentations have distinct correlates with important implications for treatment planning. In a study of adults with ARFID, those who feared aversive consequences had greater gastrointestinal sensitivity than other adults with ARFID, and those with lack of interest in eating or food underate in response to emotional distress more frequently than those with other clinical features [23]. A recent study of 122 adult patients with ARFID who presented for medical stabilization found that fear of aversive consequences was the most common presentation [24]. These categories commonly co-occur, with 51% of patients in one study presenting with more than one clinical feature [25]. However, research describing the most common clinical features of ARFID is nascent, and further investigation of these presentations, and their overlap, is needed in larger samples.

This exploratory study examined the presence of ARFID in a large sample (n = 50,082) of adult respondents to the National Eating Disorders Association (NEDA) online screen. The screen, which is publicly available, categorizes respondents into probable eating disorder diagnostic and risk categories based on established criteria [26]. However, given that the screen does not include a validated screener for ARFID, we created ARFID screening items based on diagnostic criteria and aimed to validate their capability of detecting ARFID symptoms. Among all screen respondents over a 1 year period, we assessed the prevalence and correlates (i.e., demographics, eating disorder symptoms, BMI, suicidality, treatment-seeking, treatment status) of a positive ARFID screen compared to other eating disorder diagnostic/risk categories and individuals with low/no risk for an eating disorder. In order to generate hypotheses for future research, we included all available variables on the NEDA screen as possible correlates of a positive ARFID screen. We also examined the clinical characteristics (i.e., lack of interest in eating, sensory avoidance, fear of aversive consequences of eating) endorsed by respondents who screened positive for possible ARFID.

## Methods

### Participants and procedures

Participants were adults who completed the NEDA online eating disorder screen between January 1, 2022 and January 1, 2023. The screen is publicly available on NEDA’s website (https://www.nationaleatingdisorders.org/screening-tool) [27] and assesses demographics, eating disorder attitudes and behaviors, probable eating disorder diagnoses, BMI, suicidal ideation, and eating disorder treatment status among all respondents. Respondents were included if they were ≥ 18 years of age and in the U.S. After completing the screen, respondents were provided feedback on their probable eating disorder diagnosis or risk level and given referral information. Respondents who screened positive for a probable eating disorder on the screen were given optional additional questions about their intentions to seek treatment for an eating disorder in the future.

Through our partnership with NEDA to disseminate the Stanford-Washington Eating Disorders screen (SWED), NEDA has granted approval to our research team to analyze de-identified screen data [26]. The Washington University in St. Louis Institutional Review Board provided approval to analyze screen data from U.S. adult respondents (IRB ID: 201707076).

### NEDA screen measures

#### Demographics

Participants self-reported their age, gender identity, race, ethnicity, and income on the NEDA online screen.

#### Eating disorder symptoms and probable diagnoses

Eating disorder psychopathology and all probable eating disorder diagnoses except for ARFID were assessed via the SWED included on the NEDA online screen [28, 29]. Eating disorder behaviors and attitudes assessed included frequency of binge eating and compensatory weight control behaviors (i.e., fasting, vomiting, laxative/diuretic use, excessive exercise) over the past 3 months, presence of regular dietary restriction (< 1200 kcal/day) for weight/shape reasons, and severity of weight/shape concerns based on the Weight Concerns Scale, which is included in the SWED [30]. The SWED demonstrates good sensitivity (0.68 to 0.90) and specificity (0.79 to 0.99) [29] for most DSM-5 eating disorders [1]; however, ARFID was not included in this study, and thus the SWED has not been validated to detect ARFID.

Respondents were only shown questions assessing ARFID symptoms if they screened low risk for other eating disorders. We created the following screening items to assess possible ARFID based on diagnostic criteria: (1) “Do you struggle with a lack of interest in eating or food AND has this led to major problems for you (e.g., significant weight loss and/or nutritional problems; major impairment in functioning)?”; (2) “Do you avoid many foods because of such features as texture, consistency, temperature, or smell, AND has this led to major problems for you (e.g., significant weight loss and/or nutritional problems; major impairment in functioning)?”; and (3) “Do you avoid certain or many foods, not for a medical reason such as gluten sensitivity, but because of fear of experiencing negative consequences like choking or vomiting AND has this led to major problems for you (e.g., significant weight loss, significant nutritional problems; major impairment in functioning)?” A positive screen for ARFID was defined by endorsing any one of these questions.

Participants were categorized into one of the following probable diagnostic categories based on their responses: (1) ARFID; (2) AN; (3) clinical/subclinical bulimia nervosa (BN); (4) clinical/subclinical binge eating disorder (BED); (5) purging disorder (PD); (6) unspecified feeding or eating disorder (UFED); (7) at risk for an eating disorder; or (8) at low/no risk for an eating disorder.

#### Suicidality

Item 9 from the Patient Health Questionnaire-9 (PHQ-9) [31] was used to assess suicidal ideation over the past two weeks. This item detects suicide risk with excellent sensitivity (0.88) and good specificity (0.66) [32]. Responses were coded as a binary variable indicating presence or absence of suicidal ideation.

#### BMI

BMI (kg/m^2^) was calculated from respondents’ self-reported height and weight on the SWED.

#### Current treatment status

Participants self-reported whether they were currently in treatment for an eating disorder on the NEDA screen (possible responses: “Yes,” “No,” and “Not currently, but I have been in the past”). Responses were recoded as a binary variable (Yes/No).

#### Treatment-seeking intentions

After completing the NEDA screen, respondents who screened positive for a probable eating disorder diagnosis were shown screen feedback and referral information, followed by an optional question evaluating their intention to seek treatment for an eating disorder: “Do you intend to seek professional help and/or take any steps to address these concerns?” (response options: “Definitely not,” “Probably not,” “Probably,” and “Definitely”). Responses were recoded as a binary variable (Yes/No).

### Statistical analyses

Analyses were conducted using R version 4.1.3 [26]. Of all adults who completed the screen during the recruiting period (n = 62,680), 11,813 were removed because they were outside of the U.S. (per IRB approval), and 692 were removed due to missing height/weight (and thus a probable diagnosis could not be generated). In line with our previous studies [27], 93 respondents were excluded from analysis for the following reasons: (1) self-reported eating disorder behaviors that were above implausible values (i.e., frequency of binge eating, fasting, laxative/diuretic use, exercise, and vomiting > 500 over the course of 3 months); (2) biologically implausible weights for adults (i.e., < 60 lbs); and (3) biologically implausible heights for adults (i.e., < 48 in or > 84 in). The final analytic sample included 50,082 respondents.

The proportion of respondents who screened positive for possible ARFID on the NEDA screen was calculated. Holm-corrected chi-square tests (for categorical variables) and Tukey-corrected one-way ANOVAs (for continuous variables) were used to compare demographics (i.e., age, gender, race, ethnicity, income), weight/shape concerns, eating disorder behaviors (i.e., binge eating episodes, vomiting, laxative/diuretic use, fasting, excessive exercise, dietary restriction), BMI, current treatment status, and treatment-seeking intentions between respondents with possible ARFID versus other diagnoses. Results were deemed statistically significant at a conservative cutoff of *p* < 0.01 and significant results were followed with pairwise comparisons.

To examine the clinical characteristics of ARFID, we calculated the proportion of respondents with possible ARFID who endorsed each of the following items: (1) lack of interest in eating or food, (2) avoidance of foods due to texture, consistency, temperature, or smell, and (3) avoidance of foods due to fear of experiencing negative consequences like choking or vomiting. The proportion of respondents who endorsed each possible combination of these items was also calculated.

## Results

### Prevalence of possible ARFID

Of 50,082 respondents who completed the NEDA screen, 2378 (4.7%) screened positive for possible ARFID (Table 1). Other probable diagnoses included AN (4.5%), clinical/subclinical BN (29.8%), clinical/subclinical BED (14.6%), PD (1.7%), UFED (30.3%), at risk for an eating disorder (9.6%), and low/no risk for an eating disorder (4.7%).

**Table 1:** Prevalence of probable eating disorder diagnoses and risk

Probable Diagnosis	N (%)
ARFID	2378 (4.7%)
AN	2273 (4.5%)
Clinical/subclinical BN	14,911 (29.8%)
Clinical/subclinical BED	7302 (14.6%)
UFED	15,172 (30.3%)
Purging disorder	868 (1.7%)
At risk for an eating disorder	4801 (9.6%)
Low/no risk for an eating disorder	2377 (4.7%)

Percentages reflect the proportion of individuals with each probable diagnosis out of all screen respondents

### Comparing demographics between respondents with possible ARFID and other eating disorders

Table 2 compares demographic characteristics between adult respondents with possible ARFID and respondents in other eating disorder diagnostic/risk categories. Respondents with possible ARFID tended to be younger than all other diagnostic/risk categories except those with AN (56.7% of those with ARFID were aged 18–24 compared to 62.0% of those with AN) (*p* < 0.01, Cramer’s *V* = 0.08). Respondents with possible ARFID were more likely to be male (except for those with low/no ED risk), more likely to have lower household income, less likely to be White, and more likely to be Black or African American than all other diagnostic/risk groups (*p*s < 0.01, Cramer’s *V*s = 0.04–0.05). They were more likely to be Hispanic/Latino than all diagnostic/risk groups except those with BN (*p* < 0.01, Cramer’s *V* = 0.04).

**Table 2:** Demographics by probable eating disorder diagnosis or risk level

	ARFID	AN	BN	BED	PD	UFED	At risk	Low/no risk	Significance
*Age*^*a*^*, n (%)*									*p* < 0.01, *V* = 0.08
18–24	1348 (56.7%)	1410 (62.0%)	6548 (43.9%)	1739 (23.8%)	392 (45.2%)	6368 (42.0%)	1741 (36.3%)	892 (37.5%)	
25–34	703 (29.6%)	425 (18.7%)	4534 (30.4%)	2383 (32.6%)	215 (24.8%)	4318 (28.5%)	1332 (27.7%)	642 (27.0%)	
35–44	171 (7.2%)	212 (9.3%)	2054 (13.8%)	1518 (20.8%)	145 (16.7%)	2105 (13.9%)	761 (15.9%)	347 (14.6%)	
45–54	66 (2.8%)	116 (5.1%)	1064 (7.1%)	903 (12.4%)	75 (8.6%)	1314 (8.7%)	515 (10.7%)	251 (10.6%)	
55–64	45 (1.9%)	67 (2.9%)	521 (3.5%)	537 (7.4%)	30 (3.5%)	710 (4.7%)	291 (6.1%)	165 (6.9%)	
65 +	45 (1.9%)	43 (1.9%)	190 (1.3%)	222 (3.1%)	11 (1.2%)	357 (2.4%)	161 (3.4%)	80 (3.4%)	
*Gender*^*b*^*, n (%)*									*p* < 0.01, *V* = 0.05
Female	1808 (76.0%)	2014 (88.6%)	12,894 (86.5%)	6494 (88.9%)	775 (89.3%)	12,551 (82.7%)	4149 (86.4%)	1808 (76.4%)	
Male	349 (14.7%)	99 (4.4%)	1273 (8.5%)	548 (7.5%)	41 (4.7%)	1712 (11.3%)	413 (8.6%)	454 (19.2%)	
Non-binary	147 (6.2%)	87 (3.8%)	502 (3.4%)	167 (2.3%)	36 (4.1%)	614 (4.0%)	149 (3.1%)	53 (2.2%)	
Other	66 (2.8%)	57 (2.5%)	221 (1.5%)	81 (1.1%)	14 (1.6%)	260 (1.7%)	74 (1.5%)	50 (2.1%)	
*Race*^*c*^*, n (%)*									*p* < 0.01, *V* = 0.04
White	1793 (75.4%)	1794 (78.9%)	11,532 (77.3%)	6192 (84.8%)	700 (80.6%)	12,152 (80.1%)	3736 (77.8%)	1844 (78.5%)	
Black	151 (6.3%)	49 (2.2%)	726 (4.9%)	242 (3.3%)	26 (3.0%)	682 (4.5%)	223 (4.6%)	128 (5.5%)	
Asian	108 (4.5%)	141 (6.2%)	695 (4.7%)	188 (2.6%)	37 (4.3%)	626 (4.1%)	247 (5.1%)	123 (5.2%)	
Multiracial	152 (6.4%)	139 (6.1%)	735 (4.9%)	287 3.9%)	46 (5.3%)	691 (4.6%)	221 (4.6%)	97 (4.1%)	
Other	153 (6.5%)	105 (4.6%)	1105 (7.4%)	346 (4.8%)	55 (6.3%)	904 (6.0%)	311 (6.5%)	156 (6.6%)	
*Ethnicity*^*d*^*, n (%)*									*p* < 0.01, *V* = 0.04
Hispanic	326 (13.7%)	234 (10.3%)	2277 (15.3%)	769 (10.5%)	108 (12.4%)	1801 (11.9%)	605 (12.6%)	271 (11.5%)	
Non-Hispanic	2039 (85.7%)	2009 (88.4%)	12,567 (84.3%)	6504 (89.1%)	757 (87.2%)	13,290 (87.6%)	4152 (86.5%)	2083 (88.5%)	
*Income*^*e*^*, n (%)*									*p* < 0.01, *V* = 0.04
< $20 k	487 (20.5%)	394 (17.3%)	2347 (15.7%)	764 (10.5%)	156 (18.0%)	2380 (15.7%)	624 (13.0%)	318 (13.9%)	
$20 k-59,999	829 (34.8%)	640 (28.1%)	4557 (30.6%)	2066 (28.3%)	250 (28.8%)	4271 (28.2%)	1211 (25.2%)	554 (24.2%)	
$60 k-99,999	486 (20.4%)	479 (21.1%)	3288 (22.1%)	1903 (26.0%)	189 (21.7%)	3427 (22.5%)	1086 (22.6%)	557 (24.3%)	
$100 k-150 k +	499 (21.0%)	643 (28.2%)	4322 (29.0%)	2379 (32.6%)	249 (28.7%)	4599 (30.3%)	1674 (34.9%)	861 (37.6%)	

Pairwise comparisons were conducted following significant Chi-square tests

^a^Respondents with possible ARFID were more likely to be aged 18–24 than all other diagnostic/risk categories except those with AN

^b^Respondents with possible ARFID were more likely to be male and less likely to be female than all other diagnostic/risk categories except those with low/no ED risk

^c^They were less likely to be White, and more likely to be Black or African American than all other diagnostic/risk groups

^d^They were also more likely to be Hispanic/Latino than all diagnostic/risk groups except those with BN

^e^They were more likely to have lower household income (< $20,000 and $20,000–59,999) than all other diagnostic/risk categories

### Comparing eating disorder symptoms, suicidality, and current treatment status between respondents with possible ARFID and other eating disorder diagnostic/risk categories

Table 3 compares eating disorder attitudes and behaviors, suicidal ideation, BMI, current treatment status, and treatment-seeking intentions between respondents with possible ARFID and those in other diagnostic/risk categories. On average, respondents with possible ARFID had lower weight/shape concerns than other diagnostic/risk categories (*p* < 0.001, *η*^*2*^ = 0.44) and lower dietary restriction for weight/shape reasons than all other diagnostic/risk categories except those with BED and low/no ED risk (*p* < 0.001, Cramer’s *V* = 0.40). They had lower binge eating than those with probable AN, BN, BED, and UFED, lower vomiting and laxative/diuretic use than those with probable AN, BN, and PD, and lower fasting and excessive exercise than those with probable AN, BN, PD, and UFED (*p*s < 0.01, *η*^*2*^*s* = 0.07–0.16).

**Table 3:** Rates of eating disorder symptoms, suicidality, treatment-seeking intentions, and current treatment status by probable eating disorder diagnostic/risk category

	ARFID	AN	BN	BED	PD	UFED	At risk	Low/No risk	Significance	Pairwise Comparisons
Weight/shape concerns^a^, M (SD)	24.1 (12.5)	78.2 (15.1)	78.3 (13.4)	72.2 (15.2)	81.5 (17.3)	61.8 (21.2)	65.0 (16.1)	28.2 (13.6)	*p* < 0.001, η^2^ =0 .44	AN, BN, BED, PD, UFED, at risk, low/no risk > ARFID
Binge eating, M (SD)	0.2 (0.6)	6.54 (18.3)	24.0 (30.4)	30.3 (33.1)	0.0 (0.0)	9.6 (21.5)	0.5 (0.8)	0.5 (1.6)	*p* < 0.001, η^2^ = 0.16	AN, BN, BED, UFED > ARFID
Vomiting, M (SD)	0.0 (0.2)	9.7 (38.7)	8.0 (24.4)	0.1 (0.3)	32.1 (94.2)	1.1 (6.7)	0.0 (0.2)	0.1 (1.2)	*p* < 0.001, η^2^ = 0.07	AN, BN, PD > ARFID
Laxative/diuretic use, M (SD)	0.0 (0.2)	6.7 (19.5)	5.0 (18.4)	0.0 (0.2)	25.0 (40.1)	0.8 (5.6)	0.1 (0.3)	0.0 (0.2)	*p* < 0.01, η^2^ = 0.10	AN, BN, PD > ARFID
Fasting, M (SD)	0.1 (0.3)	14.6 (25.3)	10.0 (17.9)	0.1 (0.4)	17.5 (27.0)	6.1 (15.7)	0.2 (0.5)	0.1 (0.8)	*p* < 0.001, η^2^ = 0.09	AN, BN, PD, UFED > ARFID
Excessive exercise, M (SD)	0.1 (0.3)	15.2 (28.0)	9.7 (19.5)	0.1 (0.3)	17.1 (28.9)	5.8 (16.8)	0.1 (0.4)	0.1 (0.8)	*p* < .001, η^2^ = .08	AN, BN, PD, UFED > ARFID
BMI, M (SD)	21.4 (5.4)	17.1 (1.3)	29.1 (8.6)	35.1 (9.7)	24.9 (6.3)	28.1 (8.9)	27.1 (8.7)	25.5 (6.6)	*p* < 0.001, η^2^ = 0.16	BN, BED, UFED > ARFID > AN
Dietary restriction^b^, n (%)	610 (25.7%)	2273 (100%)	7859 (52.7%)	1197 (16.4%)	736 (84.8%)	6867 (45.3%)	1983 (41.3%)	175 (7.4%)	*p* < 0.001, *V* = 0.40	AN, BN, PD, UFED, at risk > ARFID > BED, low/no risk
Suicidal ideation, n (%)	830 (34.9%)	1285 (56.5%)	6814 (45.7%)	2275 (31.2%)	476 (54.8%)	5357 (35.3%)	1366 (28.5%)	200 (8.4%)	*p* < 0.001, *V* = 0.20	AN, BN, PD > ARFID > BED, at risk, low/no risk
Treatment-seeking intentions, n (%)	25 (47.2%)	16 (34.0%)	152 (55.1%)	70 (66.7%)	6 (46.2%)	128 (46.4%)	35 (35.0%)	–	*p* < 0.05, *V* = 0.19	BN, BED > ARFID > AN, PD, UFED, at risk
Currently in treatment, n (%)	57 (2.4%)	134 (5.9%)	415 (2.8%)	157 (2.2%)	61 (7.0%)	409 (2.7%)	114 (2.4%)	–	*p* < 0.01, *V* = 0.06	AN, PD > ARFID

Eating disorder behaviors refer to frequency of behaviors over the past 3 months

^a^The possible range of scores on the Weight Concerns Scale was 0–100

^b^The dietary restriction item in this study assessed dietary restriction for weight/shape reasons

Respondents with possible ARFID had lower average BMI than those with BN, BED, and UFED and lower BMI than those with possible AN (*M* of 21.4 among those with possible ARFID versus 17.1 among those with AN, *p* < 0.001, *η*^*2*^ = 0.16), and lower suicidal ideation than those with AN, BN, PD, and low/no risk, but higher suicidal ideation than those with BED or at risk (*p* < 0.001, Cramer’s *V* = 0.20). Finally, those with ARFID had lower odds of currently being in treatment than those with AN or PD (*p* < 0.01, Cramer’s *V* = 0.06). At a 5% significance level, they had lower treatment-seeking intentions than those with BN or BED but higher intentions than those with AN, PD, UFED, or at risk (*p* < 0.05, Cramer’s *V* = 0.19).

### Clinical features of possible ARFID

Table 4 describes the prevalence of each clinical characteristic endorsed by respondents with possible ARFID. The most common clinical feature was lack of interest in eating (80.0%), followed by food sensory avoidance (55.4%) and avoidance of food due to fear of aversive consequences (30.8%). Overlap between clinical features was common, with lack of interest in eating and food sensory avoidance being the most common combination (49.0%), followed by lack of interest in eating only (26.6%), lack of interest in eating and avoidance due to fear of aversive consequences (25.7%), food sensory avoidance and avoidance due to fear of aversive consequences (23.4%), and presence of all three features (21.2%).

**Table 4:** Clinical characteristics endorsed by respondents with a positive ARFID screen

Clinical feature endorsed	N (%)
Lack of interest in eating	1902 (80.0%)
Food sensory avoidance	1317 (55.4%)
Avoidance due to fear of negative consequences	732 (30.8%)

Combinations of clinical features endorsed	N (%)
Lack of interest in eating only	632 (26.6%)
Food sensory avoidance only	101 (4.2%)
Avoidance due to fear of negative consequences only	71 (3.0%)
Lack of interest in eating and food sensory avoidance	1165 (49.0%)
Lack of interest in eating and avoidance due to fear of negative consequences	610 (25.7%)
Food sensory avoidance and avoidance due to fear of negative consequences	556 (23.4%)
Lack of interest in eating and food sensory avoidance and avoidance due to fear of negative consequences	505 (21.2%)

## Discussion

This study examined the prevalence, demographic and psychopathological correlates, and clinical characteristics of possible ARFID among adult respondents to a widely disseminated online eating disorders screen. In this study, a positive ARFID screen was slightly more prevalent than a positive AN screen (4.7% vs. 4.5%), highlighting that a significant proportion of adults with disordered eating likely meet criteria for ARFID. Previous population-based surveys in Australia have estimated comparable point prevalences of ARFID and AN (0.3% and 0.4%, respectively) [3]. Of note, respondents to the NEDA online screen represent a high-risk sample for eating disorders.

In line with prior research in treatment samples, we found that NEDA screen respondents who screened positive for possible ARFID were more likely to be male compared to respondents in all other probable eating disorder diagnostic/risk categories [15]. Several previous studies have found that patients with ARFID tend to be younger than those with other eating disorders, including AN [7, 15], possibly due to the earlier age of onset of ARFID (i.e., typically during childhood) compared to other eating disorders [1]. Similarly, in this study, both respondents with positive AN and ARFID screens were significantly younger than those in other probable diagnostic/risk categories. However, respondents with probable AN were the youngest, with a larger proportion of respondents aged 18–24 than those with possible ARFID, whereas those with ARFID were more likely than those with AN to be aged 25, 27–35. One possible explanation for individuals with probable ARFID in our sample being younger than those with AN on average may be related to the characteristics of individuals who complete the NEDA screen. For instance, perhaps younger individuals who seek out and complete an online ED screen are more likely to experience the weight/shape concerns and associated distress that is consistent with an AN presentation.

We found that respondents who screened positive for possible ARFID were less likely to be White relative to respondents in all other diagnostic/risk groups. One previous study of a clinical sample of adults found the same pattern of results [20], whereas a study of a treatment-seeking community sample did not [33]. We also found that respondents with possible ARFID were more likely to be Hispanic/Latino than those in all diagnostic/risk groups except for BN, which was also found in the study of the treatment-seeking community sample [33]. To our knowledge, this was the first study to examine household income as a correlate of ARFID, and we found that those with a positive ARFID screen were more likely to have lower household income compared to all other probable diagnostic/risk categories. These results could suggest that ARFID may more broadly affect adults across demographics compared to other eating disorders. Alternatively, our findings may suggest that a larger proportion of individuals with ARFID symptoms across demographic groups seek out the NEDA screen due to lack of screening or access to treatment for ARFID. Given the demographic differences between those who screened positive for ARFID relative to other diagnostic/risk groups, another possibility is that individuals who engage in restriction for reasons that are not captured by the general ED screening items (e.g., drive for muscularity) were more likely to endorse the ARFID items.

We examined the eating disorder symptomatology of respondents who screened positive for possible ARFID. In line with some previous research, respondents with a positive ARFID screen had a lower BMI than those in most other probable eating disorder/risk categories, except for AN [3, 13, 17]. Of note, some previous work has found that the BMI of patients with ARFID was comparably low relative to those with AN [16]. We also found that respondents with a positive ARFID screen had lower dietary restriction for weight/shape reasons, weight/shape concerns, binge eating, compensatory weight control behaviors than those in most other diagnostic/risk categories, as expected. However, 26% of those with possible ARFID according to our screen items endorsed dietary restriction for weight/shape purposes. Given that ARFID is characterized by dietary restriction that is not attributed to weight/shape concerns (which distinguishes it from AN), these results may indicate that our screening items lack sensitivity to detect a probable ARFID diagnosis. Because respondents who screened low/no risk for other eating disorders were shown the ARFID items, it is possible that respondents who reported dietary restriction and weight/shape concerns in the absence of low BMI (i.e., symptoms resembling an atypical AN presentation) in addition to endorsing food avoidance on the ARFID items were categorized as having a positive ARFID screen. On the other hand, results may suggest that respondents with probable ARFID may experience weight/shape concerns and engage in weight/shape-driven restriction to a degree that does not rise to the level of clinical impairment. Because the NEDA screen does not identify atypical AN and because we did not compare ARFID characteristics between those with probable ARFID and those with other probable eating disorders, we are unable to draw conclusions about these results. Given these measurement challenges, it is critical for future research to develop and validate brief screening measures for ARFID while accounting for these limitations. Recent research has shown preliminary support for the nine-item ARFID screen (NIAS) [34], which measures restrictive eating associated with appetite, fear, and picky eating [35]. However, to date, no brief screening tool for ARFID has been rigorously validated. Our study aimed to estimate the prevalence of possible ARFID with three brief items in lieu of other available screening tools. However, results indicate that our items have limitations, and thus, results of this study should be considered tentative.

Thirty-five percent of respondents with a positive ARFID screen in our sample reported suicidal ideation. This rate was lower than that of those with AN, BN, and PD, but higher than those with BED, at risk for an eating disorder, or those with low/no risk. Prior research in this area has been mixed, with one study finding lower suicidal ideation among youth with ARFID versus AN [19] and two others finding no differences in rates of suicidal ideation between adults and adolescents with ARFID versus other eating disorders [20, 36]. Nonetheless, previous literature has established that restrictive eating, which is a core characteristic of ARFID, is associated with higher risk of suicidal ideation [37]. It has been theorized that frustrations due to inconclusive assessment that patients often receive in medical facilities for their gastrointestinal symptoms, as well as low body trust (e.g., due to experiences of choking or vomiting) could increase hopelessness and suicidal ideation among those with ARFID [20, 38]. Moreover, one previous study found that youth with acute ARFID symptoms had higher suicidal ideation and/or self-harm than those with chronic symptoms, suggesting that symptom duration is an important factor [25]. Taken together, adults with ARFID may represent a high-risk group for suicide, warranting further research and routine screening in this population.

Although a sizable proportion (47%) of screen respondents with possible ARFID expressed intentions to seek treatment for an eating disorder, few (2%) had actually initiated treatment. In comparison to those with other probable eating disorders, respondents with possible ARFID were less likely to be in treatment for an eating disorder than those with AN or PD, and they had lower treatment-seeking intentions than those with BN or BED but higher intentions than those with AN, PD, UFED, or at risk. Although no previous research to our knowledge has compared eating disorder treatment rates among patients with ARFID relative to other eating disorders, it has been documented that patients with ARFID often present to medical facilities (i.e., non-mental health facilities) for treatment to manage gastrointestinal symptoms [39]. However, despite the fact that 13–40% of adults presenting to neurogastroenterology clinics report symptoms consistent with ARFID, existing assessment measures have not been validated for this key population [40, 41], which can lead ARFID to go undetected [12]. In fact, prior research has indicated that patients with ARFID have a longer duration of illness before receiving medical intervention relative to those with other types of eating disorders [42]. It should also be noted that probable ARFID was most prevalent among those with low income in our sample, a population that may be less likely to access treatment, and this problem may be exacerbated by the lack of available treatments for ARFID. Efforts to develop and disseminate effective interventions for ARFID are needed in order to address the treatment gap and serve those most frequently affected by ARFID and who may lack access to treatment.

Most (80%) respondents with possible ARFID in this study reported a lack of interest in eating, followed by food sensory avoidance (55%) and avoidance of food due to fear of aversive consequences (31%). Although prior research has not established the most prevalent presentation, all three presentations are common in clinical settings [22]. Some literature suggests that the most prominent clinical profiles of ARFID may vary significantly across populations. For example, one study found that adult neurogastroenterology patients with ARFID most frequently reported fear of gastrointestinal symptoms as motivation for their dietary restriction [40]; fear of choking or vomiting was also found to be prevalent among adolescents in a partial hospitalization program [43]. In line with previous literature [25, 44], overlap between clinical features was common in our study, with 49% of those with possible ARFID reporting both lack of interest in eating and food sensory avoidance. Previous work has theorized that the clinical presentations of ARFID are not mutually exclusive, and instead can be conceptualized as dimensions on which each patient can be high or low [22]. Although research in this area is still emerging, recent work has indicated that each clinical presentation has unique psychopathological correlates and may be differentially associated with illness duration and severity [5]. For example, food sensory sensitivity is often a longstanding condition and has been associated with greater likelihood of comorbid neurodevelopmental, disruptive, and conduct disorders, anxiety, obsessive–compulsive, and trauma-related disorders, and depressive and bipolar-related disorders; severity of fear of aversive consequences has been associated with greater likelihood of comorbid depressive and bipolar-related disorders [44, 45]. Additional research is needed to inform how treatments can best address each clinical profile of ARFID and its associated psychopathology.

A strength of this study was that we leveraged a widely disseminated online eating disorders screen in order to conduct the largest study of ARFID prevalence to date. This study also examined key correlates of ARFID (e.g., income) that had previously not been studied. Limitations included that our ARFID screening items have not been previously validated and thus results should be considered tentative. In addition, this sample was comprised of individuals who voluntarily completed a screen and thus was not nationally representative. Further, because our screen followed DSM-5 criteria and only showed ARFID items to individuals who did not screen positive for another ED, it may not have captured more nuanced presentations of symptoms. Although the DSM-5 has proposed the three presentations of ARFID presented in this study, additional presentations of ARFID may exist and future work is needed to continue investigating possible additional presentations. In addition, future studies should explore whether the three recognized ARFID presentations have distinct patterns of comorbidity and etiologies. Finally, our study did not include data on forms of general psychopathology other than suicidality, which represents an important direction for future studies examining correlates of ARFID. This study highlighted several other important areas for future research, including examining prevalence of ARFID across racial, ethnic, and income groups, advancing research on the clinical profiles of ARFID and their treatment implications, and addressing the treatment gap among individuals with ARFID.

## Conclusions

Findings from this study indicated that, among adult respondents to a publicly available online eating disorders screen, a positive ARFID screen was more common among individuals who were younger, male, non-White, Hispanic, and lower income relative to those in other eating disorder diagnostic/risk categories. Results also suggested that individuals with possible ARFID frequently reported suicidal ideation and were rarely in treatment for an eating disorder. Further research is urgently needed to improve advances in the assessment and treatment of ARFID in order to prevent prolonged illness duration.

## Data Availability

The data that support the findings of this study are available from the National Eating Disorders Association, but restrictions apply to the availability of these data, which were used under approval for the current study, and so are not publicly available. Data are however available from the authors upon reasonable request and with permission of the National Eating Disorders Association.

## Abbreviations

AN: Anorexia nervosa
ARFID: Avoidant-restrictive food intake disorder
BED: Binge eating disorder
BN: Bulimia nervosa
NEDA: National Eating Disorders Association
SWED: Stanford-Washington eating disorders screen
UFED: Unspecified feeding or eating disorder
